# Drei Jahre Gallenstein Chirurgie

**Published:** 1910-06

**Authors:** 


**Drei Jahre Gallenstein Chirurgie.** Von Prof. Dr. Hans Kehr, Dr. Liebold, und Oberarzt Dr. Neuling. Pp. xiv, 722. München: J. F. Lehmann. 1908.

This book is divided into a first part of 492 pages, giving the clinical histories of 300 patients submitted to laparotomy for affections of the biliary system, mostly cases of gall-stone, and a second part of 200 pages, in which the preceding cases are analysed and classified, and the whole subject of gall-stone surgery is thoroughly and scientifically considered. The case-records are exceedingly well indexed, so that the reader can rapidly refer to those which illustrate any variety of operative procedure he particularly wishes to investigate, and nearly all types of operative procedure are exemplified.

The case-records are not mere abstracts, but give in full all important details, with the exception of the permanent after-results. The latter cannot be given, for all the operations were performed in the three years 1904 to 1906, and too little time has elapsed for the permanency of most of the results to be fairly tested.

We shall not attempt here to summarise in any way the author's conclusions, but we have no hesitation in saying that in our opinion the book is an exceedingly valuable addition to the literature of the subject with which it deals, and we strongly advise all surgeons who are called upon to deal with gall-bladder affections to give it their earnest attention.

In our opinion the reader will find it best to begin by reading the general account of the cases and of gall-stone surgery in Part II, and to refer to the case-records of Part I, as occasion demands, for examples of the application of the various operative methods described.

